# Genetic tests as the strongest motivator of cooperation between participants and biobanks–Findings from cross-sectional study

**DOI:** 10.3389/fgene.2024.1321690

**Published:** 2024-05-17

**Authors:** Anita Majchrowska, Jakub Pawlikowski, Jarosław Sak, Blanka Świerczyńska, Magdalena Suchodolska

**Affiliations:** ^1^ Department of Humanities and Social Medicine, Medical University of Lublin, Lublin, Poland; ^2^ Faculty of Medicine, Medical University of Lublin, Lublin, Poland

**Keywords:** biobanking, human biological material, motivations, participation, genetic tests

## Abstract

**Introduction:**

The development of the scientific potential linked with biobanking and research on human biological material is highly dependent on the willingness of potential donors to cooperate with entities that collect the material. For this reason, it is crucial to identify the circumstances and factors that may encourage potential participants to donate their biological material. In particular, knowledge of the motivational factors that can be modified by the persons managing a biobank may prove notably important for shaping the organizational and communication policy of the biobank and other scientific institutions.

**Material and methods:**

The research was carried out on a group of 1,100 people over 18 years of age representing the adult population of Poland in 2021.

**Results:**

More than half of the respondents declared their willingness to donate a blood sample for research purposes to a biobank (57.8%). The most often indicated incentives among the factors supporting the donation of biological material were offers of: obtaining the results of genetic tests predicting the risk of diseases (77.1%), blood tests (71.3%), the possibility of obtaining a small remuneration (64.6%) and the carrying out of genetic ancestry tests (60.4%).

**Conclusion:**

Offering the possibility of performing additional diagnostic tests, especially genetic tests, may significantly increase the willingness of potential donors to cooperate with biobanks and other entities collecting human biological material for the purpose of scientific research. However, attention should also be paid to the challenges and risks linked with respecting the privacy and autonomy of research participants.

## 1 Introduction

Biobanking and testing on human biological material (hereinafter: HBM) have great potential for finding new diagnostic, therapeutic and preventive methods, while at the same time, entailing low health risks for participants (when compared to clinical trials on human subjects). The conduct and effectiveness of research on HBM depend not only on the correct organization of biobanks, on having advanced laboratory infrastructure, experienced staff and maintaining high quality samples, but also on effective cooperation with potential research participants. Potential participants are persons who have not yet donated their samples for research purposes but it is possible to encourage them to participate in biobanking by effective communication The low willingness of potential participants to cooperate and the small number of samples translate onto insufficient use of the potential that HBM research offers. For this reason, it is crucial to recognize the circumstances and factors that may positively affect the willingness of potential donors to donate their material. Particularly important factors and circumstances may be those that the research managers may influence and thus shape the organizational and communication policies of biobanks and other HBM researching institutions accordingly.

Current research results indicate that the willingness of research participants to cooperate with biobanks may be significantly modified depending on a number of factors and circumstances. Both the individual psychosocial factors of donors and the organizational and social circumstances in which the biobank operates play a role in their attitudes and decisions (Domaradzki and Pawlikowski, 2019). The willingness to cooperate with biobank may be significantly influenced by psychological and social factors such as: trust, preferred values, level of knowledge, social involvement, age, education, material status, place of residence or having offspring (Goddard et al., 2009; Melas et al., 2010; Al-Jumah and Abolfotouh, 2011; Ma et al., 2012; Lewis et al., 2013; Ahram et al., 2014; Pawlikowski et al., 2022). Some studies also draw attention to circumstances that discourage potential participants from donating samples, such as the risk of privacy violations (especially in the case of identifiable genetic analyses of participants), the risk of stigmatisation (e.g., in the case of the use of identifiable samples for research on mental disorders), the cooperation of public entities collecting material with entities commercially using material and data (Schwartz et al., 2001; Simon et al., 2011), and even the distance from the place of residence of potential donors to the seat of a biobank (Porteri et al., 2014).

What proves particularly important from the perspective of developing effective cooperation with potential research participants, is to examine those motivational factors that depend on entities collecting biological material and which can be modified within their organization, management and use of accumulated resources. These may include, for example, the offer of additional laboratory tests in the field of biochemical and genetic analysis for donors of material, the manner and scope of making samples available to other entities, the directions of scientific research in which samples and data are used, as well as the donor communication policy of the biobank, including information on the results of scientific research carried out on samples. Such knowledge may be embedded in the strategy of responsible management and effective communication with potential research participants and the social environment.

The objective of our research was to identify/analyze the social perception of factors encouraging or discouraging the donation of biological material to a biobank in the Polish population. We focused on those elements and circumstances that did not result from the subjective characteristics of the participants (which was the subject of other research), but were related to the activities of biobanks, their organization, management methods and their cooperation with donors. Identifying the factors motivating participants to donate their biological material, which the biobanks may propose, will allow developing a catalogue of effective incentives to ensure effective cooperation with potential donors.

The research was conducted as part of a task dedicated to the ethical, legal and societal implications of biobanking within the remits of a project of establishing the Biobanking and BioMolecular resources Research Infrastructure–European Research Infrastructure Consortium (BBMRI.ERIC) (Witoń et al., 2017).

## 2 Materials and methods

### 2.1 Study participants

The research was carried out on a group of 1,100 people over 18 years of age representing the adult population of Poland in 2021. In order to determine the size and structure of the sample, data of the Statistics Poland regarding the population aged 18 and over (as of 31.12.2020) were used. After setting the permissible statistical error margin at the level of approx. 3% (for the confidence level a = 0.95, the distribution of responses = 0.5 and the size of the adult population equal to 32,386 679), we calculated that the research sample should consist of 1,100 inhabitants of Poland. The selection of respondents was determined on the basis of their being representative of the Polish population in the following areas: sex of respondents (100% compliance with calculations based on the Local Data Bank [LDB]), age of respondents (maximum deviation of 2% from calculations based on the LDB), the number of respondents in a given voivodeship calculated on the basis of the population distribution throughout the country (100% compliance with calculations based on the LDB), place of residence (maximum deviation of 1% from calculations based on the LDB), level of education (maximum deviation of 3% from calculations based on the LDB) (Table 1).

**TABLE 1:** Sample characteristics.

Variables		N	%
Age			
	18–29	177	16
	30–44	317	29
	45–59	254	23
	Over 60	352	32
Sex	Women	574	52
	Men	526	48
Education	Primary or vocational	464	42.2
	Secondary	379	34.4
	High	257	23.4
Place of living	Village	441	40.1
	City to 50.000 residents	265	24.1
	City from 50.000 to 100.000 residents	186	16.9
	City over 100.000 residents	208	18.9
Self-assessment of material conditions	Very bad	9	0.8
	Bad	31	2.8
	Rather bad	178	16.2
	Rather good	528	48.0
	Good	272	24.7
	Very good	82	7.5
Self-assessment of health conditions	Very bad	13	1.1
	Bad	26	2.3
	Rather bad	195	17,6
	Rather good	484	43.9
	Good	268	24.3
	Very good	112	10.8

## 3 Data collection

The data comes from quantitative cross-sectional survey-based research carried out in 2021, using a mixed-mode survey technique comprising 60% Computer-Assisted Personal Interview (CAPI) and 40% Computer Assisted Telephone Interview (CATI). The high share of CATI technique was necessitated by the conditions of the COVID-19 pandemic that hindered direct contact with the respondents. In the research, a standardized questionnaire was used, which had been previously verified in a pilot study.

The respondents were selected using the “random route” method as a default method (employing the computer-assisted personal interview [CAPI] technique. Participation in the study was voluntary. From the starting point (first address number on the selected street), the interviewer visited every third apartment (apartment/single-family house), until a maximum of three respondents to the survey were found in the street or until the pool of addresses at which respondents meeting the criteria for participation in the survey could be exhausted. The maximum number of people from a single location is nine respondents in locations with up to 100 thousand inhabitants and 15 respondents in locations with over 100 thousand inhabitants. Gaps in the metrical data were filled in by the computer-assisted telephone interview [CATI] technique, using a number generator to draw telephone numbers from a database of active numbers issued by Polish landline and mobile operators. The structure of the studied group in terms of sociodemographic features was consistent with the structure of the Polish population and held a risk of statistical error of 3%. The detailed characteristics of the studied group are presented in Table 1.

## 4 Measures

Willingness to donate biological material to a biobank was measured using the following question: ‘*Please imagine that a biobank from the nearest provincial city, operating at a medical university, asks you to donate a blood sample for research. Approximately* 30 ml *of blood (three large tablespoons) will be drawn, and an interview will take place regarding health- and disease-related issues, such as lifestyle (e.g., eating habits, exercise, use of stimulants, sleep), environment, drug use, medical history, etc. The collected samples and data will then be made available to scientists for research in an anonymised form (i.e., the donor cannot be identified). Would you give a blood sample to a biobank in the situation described above?’* The respondents were asked to rate their willingness to donate on a five-point scale from 1 (definitely not) to 5 (definitely yes).

Motives for the donation of biological material were measured by the question: ‘*What would particularly encourage you to donate a sample of your biological material to a biobank?’*. Respondents were presented with a multiple choice of factors encouraging the donation of biological material. The respondent was to respond to each of them on a five-point scale: *“definitely yes*”, “*rather yes*”, “*difficult to say”,* “*rather no” and “definitely no”.* The list of factors that could positively affect the willingness to cooperate and the willingness to donate biological material to a biobank was elaborated on the basis of a review of the research and the opinions of six experts in ELSI in biobanking cooperating with the BBMRI. pl consortium and the Polish Biobank Network. A list of the following factors potentially encouraging cooperation was established (the first eight were assumed to be positive and the last two negative):

1. Performing genetic tests for risk of diseases;
2. Performing genetic tests on genealogy (ancestry);
3. Minor financial remuneration;
4. Reimbursement of travel expenses;
5. Possibility to learn about the results of scientific research in which your sample will be used;
6. Possibility to perform additional laboratory tests (e.g., blood sugar, cholesterol levels, *etc.*);
7. If the biobank is part of a hospital;
8. If the biobank is part of a university;
9. Cooperation of the biobank with scientific centres from abroad;
10. Cooperation of the biobank with pharmaceutical and biotechnology companies;
11. If the biobank was a private institution.

So as to gain information on respondent’s financial motivations to provide a sample of biological material, we also asked the following question: “*Should the persons donating samples of their biological material be entitled to financial remuneration?* The respondents responded to the question on a five-point scale: *“definitely yes*”, “*rather yes*”, “*difficult to say”,* “*rather no”* and “*definitely no”.* The research also included sociodemographic variables (gender, age, education, place of residence) and information on respondents’ self-assessment of their health and material conditions (on the Likert five-point scale).

## 5 Statistical analysis

Data was analyzed using the IBM SPSS Statistics v. 25 software suite. Descriptive statistics (frequency, mean, percentage and standard deviation), the t-student significance test for independent samples, r-Pearson, rho-Spearman correlation coefficients and regression with stepwise input method were applied during the analyzes. The level of statistical significance was *p* < 0.05.

For in-depth data analysis, a single factor logistic regression model was constructed using the quasi-Newton estimation method. The constructed probability model was assessed in terms of its sensitivity and specificity through analysis of the ROC (Receiver Operating Characteristics) curve.

## 6 Results

The willingness to donate blood samples for scientific research to a biobank was declared more often than not by every second respondent (by 57.8% (N = 636) of the total number of respondents, with 14.5% (N = 160) indicating the answer “definitely yes”. A small group of the assessed (16.5%, N = 182) admitted that they would not decide to donate a sample for scientific research. What is noteworthy is the large percentage of undecided people, with over ¼ of the respondents (26%, N = 282) having problems with determining an unambiguous position.

As many as 70.3% (N = 774) of all respondents confirmed that people should be entitled to financial remuneration for giving a sample of biological material (slightly less than one in three confirmed this at a decisive level). Only 9.1% (N = 100) of those taking part in the survey disagree with this statement, and every fifth (20.5%; N = 226) was unable to determine a clear position on this issue.

**FIGURE 1:**
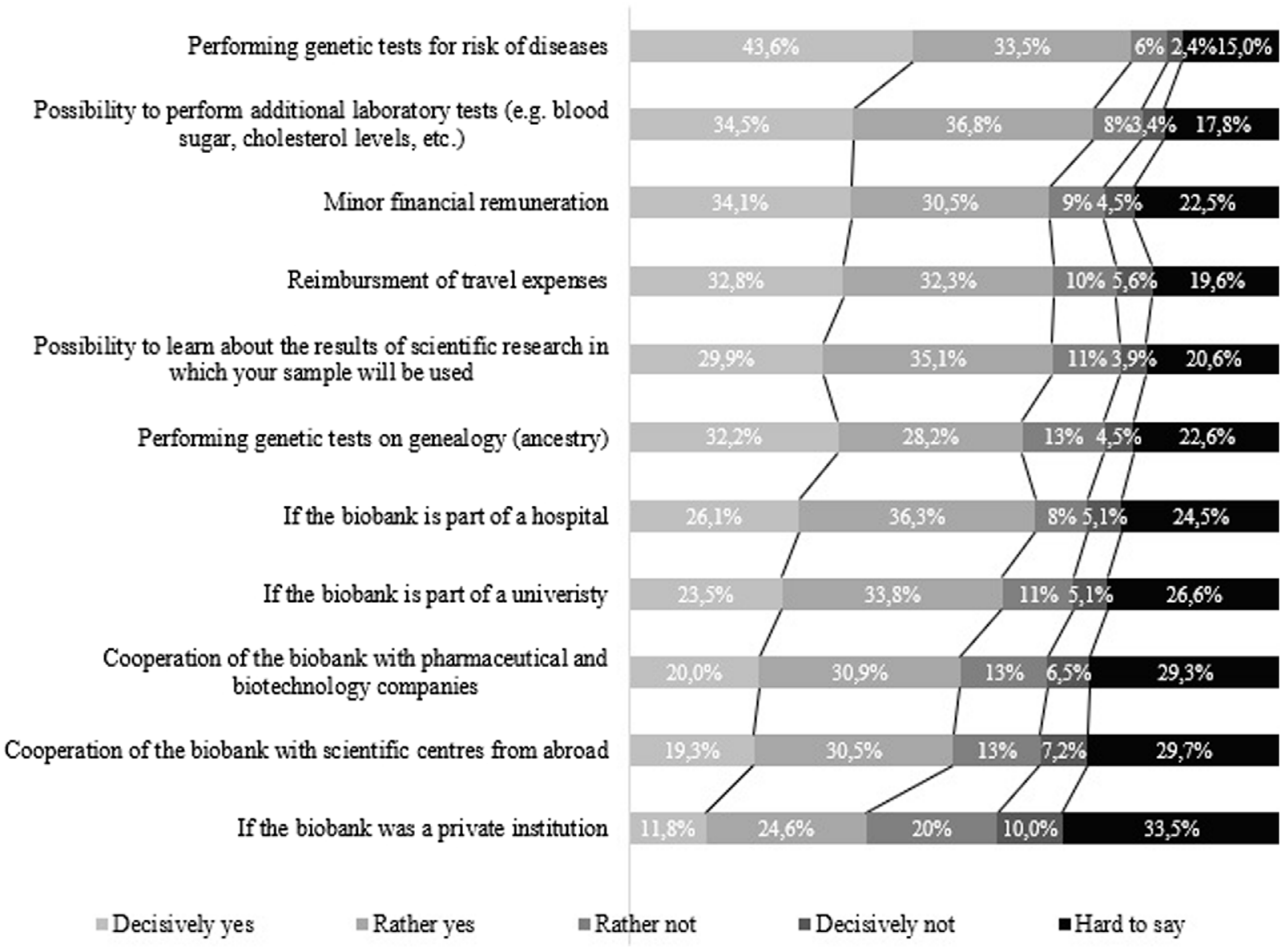
Motivating factors for donating a sample of biological material from the perspective of potential donors.

The analysis of factors that were significant for the respondents in the process of deciding on the donation of genetic material indicates that the strongest incentive for the respondents would be the possibility of carrying out genetic tests predicting the risk of diseases. This was confirmed by 77.1% (N = 849) of all the assessed, of which 43.6% (N = 480) chose the “definitely yes” answer. The possibility of additional laboratory tests, e.g., blood sugar, cholesterol levels, *etc.*, would be of an incentive for 71.3% (N = 784) of all those who took part in the survey, while learning the results of scientific research in which the sample will be used would encourage 65% (N = 715) of the respondents. What is more, genetic ancestry tests would be important for 60.4% (N = 664) of the subjects. It is worth noting the financial motivations indicated by the respondents: a small financial remuneration would constitute an incentive for 64.6% (N = 710) of them, and the reimbursement of travel costs for 65.1% (N = 716) of those who replied. Detailed results are presented in Figure 1.

The above variables were analyzed taking into account readiness to donate, sociodemographic variables and self-assessment of one’s own material situation. All the variants of incentives to donate a sample of biological material to a biobank presented to the respondents in the study showed statistically significant Spearman’s R rank correlations with the readiness to donate a blood sample to a biobank. The strongest correlations were obtained in relation to the possibility of performing additional laboratory tests, genetic tests for disease risk, learning the results of scientific research in which the respondent’s sample will be used, cooperation of the biobank with scientific centers from abroad, and the situation in which the biobank would be a part of a university or a hospital, while the weakest would be if it was a private institution (Table 2).

**TABLE 2:** Correlation analyses of the willingness to donate biological material.

			Willingness to donate a blood sample to a biobank		Age		Women N = 574		Men M = 526		U mann-whitney test	
		N	R spearman	*p*	R spearman	*p*	M	SD	M	SD	Z	p
	**Willingness to donate a blood sample to a biobank**	1,100			−0.05	0.082335	3.55	1.02	3.44	1.07	1.56	0.118
What would particularly encourage you to donate a sample of your biological material to a biobank?	Possibility to perform additional laboratory tests (e.g., blood sugar, cholesterol levels, *etc.*)	1,100	0.27	0.000001	−0.06	0.066038	3.90	1.05	3.93	1.07	−0.56	0.574
	Performing genetic tests for risk of diseases	1,100	0.29	0.000001	−0.10	0.000574	4.14	1.00	4.07	1.01	1.19	0.233
	Performing genetic tests on genealogy (ancestry)	1,100	0.25	0.000001	−0.12	0.000077	3.73	1.18	3.69	1.16	0.62	0.535
	Possibility to learn about the results of scientific research in which your sample will be used	1,100	0.31	0.000001	−0.09	0.002996	3.78	1.11	3.75	1.10	0.61	0.539
	Cooperation of the biobank with scientific centres from abroad	1,100	0.28	0.000001	−0.08	0.006859	3.36	1.16	3.48	1.14	−1.81	0.071
	Cooperation of the biobank with pharmaceutical and biotechnology companies	1,100	0.23	0.000001	−0.07	0.022112	3.40	1.14	3.49	1.14	−1.37	0.172
	If the biobank is part of a university	1,100	0.30	0.000001	0.02	0.589527	3.57	1.13	3.63	1.09	−0.67	0.500
	If the biobank is part of a hospital	1,100	0.31	0.000001	0.03	0.399856	3.69	1.12	3.71	1.06	−0.02	0.985
	If the biobank was a private institution	1,100	0.16	0.000001	−0.13	0.000011	3.01	1.14	3.16	1.15	−2.14	0.032
	Reimbursement of travel expenses	1,100	0.21	0.000001	−0.09	0.004008	3.79	1.14	3.75	1.20	0.43	0.670
	Minor financial remuneration	1,100	0.11	0.000226	−0.16	0.000001	3.83	1.14	3.79	1.12	0.91	0.361

Symbols: M-mean; SD, standard deviation; *p* < 0.05.

The analysis of sociodemographic variables revealed a weak, albeit, statistically significant correlations between the lower age of respondents and the analyzed categories of incentives. Gender significantly differentiated respondents only in one case–women would be more discouraged than men to cooperate with a biobank that would be a private institution. With regard to education, there were statistically significant differences in the readiness to donate a blood sample to a biobank (H = 10.35; *p* = 0.0057), the possibility of learning the results of scientific research (H = 6.14; *p* = 0.0464) and small financial remuneration (H = 8.43; *p* = .0148). People with higher level of education were more inclined to stress these aspects. There were no statistically significant relationships between the analyzed dependent variables and the self-assessment of respondents’ own financial situation and their place of residence.

In order to analyse the data in depth, a single factor logistic regression model was constructed using the quasi-Newton estimation method of the probability of the respondents’ readiness to donate a blood sample to a biobank, taking into account the indicated factors and circumstances. The entire studied population was divided into two fractions due to the need to define the dependent variable in binary terms. The first group (“0″) was formed from respondents who were not willing to donate a sample of their blood (“definitely not”, “rather not”) or undecided. The second group (“1″) was formed from respondents declaring their readiness to donate a blood sample.

Based on the analysis of the created regression model, it was found that the readiness to donate a blood sample to a biobank will be significantly higher if the biobank offers the possibility to perform genetic research towards the risk of diseases, as well as (although less likely) other additional analytical research, when the donors were able to learn the results of scientific research in which their sample was used, and if the biobank would cooperate with scientific centers from abroad, or it would be part of a university or a hospital (Table 3).

**TABLE 3:** Probability model of biological material donation.

The model of the probability of the respondent donating a sample of biological material to a biobank depending on the analyzed factors (N = 1,100)							
Effect		Assessment	Standard error	Wald chi-square test	GU upper 95.0%	GU lower 95.0%	p
Absolute term		−4.857	.435	124.438	−4.003	−5,712	0.00001
What would particularly encourage you to donate a sample of your biological material to a biobank?	Possibility to perform additional laboratory tests (e.g., blood sugar, cholesterol levels, *etc.*)	0.202	0.073	7.718	0.345	0.059	0.006
	Performing genetic tests for risk of diseases	0.391	0.077	25.469	0.542	0.239	0.00001
	Possibility to learn about the results of scientific research in which your sample will be used	0.213	0.072	8.703	0.355	0.071	0.003
	Cooperation of the biobank with scientific centres from abroad	0.181	0.068	7.063	0.314	0.047	0.008
	If the biobank is part of a university	0.201	0.073	7.477	0.345	0.057	0.006
	If the biobank is part of a hospital	0.178	0.074	5.874	0.322	0.034	0.016

**FIGURE 2:**
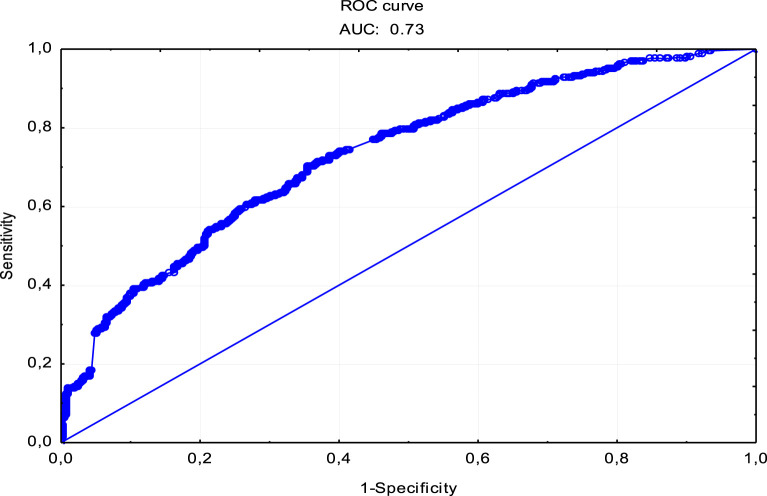
ROC curve for the single factor logistic regression model of the probability of donating a biological material sample to a biobank.

Using the ROC (Receiver Operating Characteristics) curve, we assessed the sensitivity and specificity of the constructed probability model of the respondents’ readiness to donate a blood sample to a biobank. The area under the AUC curve was determined as a measure of the accuracy and goodness of fit for this model. The obtained AUC value–0.73 indicates the appropriate accuracy of the model (Figure 2).

## 7 Discussion

The development of biobanks is not possible without an adequate number of donors of biological material that are willing to cooperate with them. It should be emphasized that without potential participants - people expressing informed consent to donate their own biological material for scientific (and sometimes also therapeutic) purposes - the idea of research using HBM loses its importance. Bearing the above in mind, it seems necessary to discover the factors increasing the probability of participation of new people in the biobanking of human biological material. It is also equally important to avoid situations that may discourage cooperation.

At the level of social perception, biobanking remains an unknown activity and a concept unknown to many people (Heredia et al., 2017). Until about a decade ago, most Europeans had never heard of or sought information on biobanks (Gaskell et al., 2013). Currently, the situation has improved slightly, but the knowledge and willingness to cooperate with biobanks varies from country to country. Undoubtedly, the decision to donate biological material results mainly from the internal motivations of donors that, according to many studies, are altruistic and related to the implementation of the common good. Indeed, study results indicate that a large proportion of donors reveal that altruistic motives and willingness to help those in need are their motivation for donation (Kettis-Lindblad et al., 2006; Overby et al., 2015; Vaz et al., 2015). Taking part in such an initiative and becoming a donor is, hence, treated as a contribution to the public good (Lemke et al., 2010; Nobile et al., 2013; Tauali et al., 2014; Dixon-Woods et al., 2017; Broekstra et al., 2022), an opportunity to bring benefit to the society as a whole (Yip et al., 2018; Raivola et al., 2019), a kind of obligation towards society (Hoeyer, 2010), and it gives a sense of personal appreciation and internal gratification due to the possibility of selfless support for the sick and suffering (Khalil et al., 2007; Ludman et al., 2010; Richter et al., 2018; Broekstra et al., 2020). In the work of Davis et al. (2019) the respondents stated that they would participate in genetic research and the donation of biological material to a biobank to help others, and, in particular, their own offspring, even if they themselves would not benefit from this activity (Pulley et al., 2008; Lhousni et al., 2020). In the study by Mezinska S. et al. (2020) respondents indicated the significant motivational role of potential benefits for relatives and people with whom they are in close relationships with (Rahm et al., 2013). The will to donate biological material is also significantly related to other psychosocial variables, such as preferred values, level of trust or the search for the meaning of life. However, the impact of biobanks on internal motives and motivations of their donors is limited, so it is important to identify those factors that biobanks can manage in order to increase the propensity to donate. Additional benefits that a biobank can offer to potential donors may constitute an important incentive to donate biological material.

In our research, we focused on selected variables related to the biobank as a motivating factor for participants to donate samples. It should be noted that the storage and access to samples by foreign researchers have been identified in some studies (Lewis et al., 2013), including those conducted in Poland (Pawlikowski, 2013), as barriers to the donation of human biological samples. However, few studies suggest that participants are receptive and supportive of sharing samples for scientific purposes (Pereira, 2013). The ethical issues arising in practice must be understood in the context of interactions between research institutions, local communities, and collaborating entities (Tindana et al., 2024).

This is an important issue in the context of the results of our own research, from which a significant percentage of people who are indecisive and do not have an unequivocal attitude towards the donation of biological material still emerges. The communication activities of biobanks should be targeted at this group of undecided people, because it will be relatively easy for them to develop a positive attitude towards biobanking. Therefore, this group should be particularly targeted by biobank activities.

In the obtained results, one of the most important factors encouraging cooperation was offering potential participants the opportunity to have genetic tests performed, both for the diagnosis of their susceptibility to diseases and for genetic ancestry research. This attitude signals how important it is for members of developed societies to access and manage genetic information, including information on genetic predisposition to diseases, on health status, as well as genealogical relationships. This can be seen as a manifestation of social genetization (Mannette, 2021), which in this case can be used to enhance the effectiveness of cooperation between potential donors and biobanks. It further seems that access to genetic research will be an even stronger motivating factor in the coming years and may become one of the important elements of cooperation between scientific entities, researchers and research participants. Considering that samples from a biobank are sometimes made available for genomic research and that genome sequencing is performed, information about such a possibility at the stage of sampling and obtaining the donor’s consent seems desirable from an ethical point of view. In this context, a particular challenge is the responsible management of genetic information held by scientific entities and researchers, and the preservation of confidentiality and privacy in the process of sharing and processing of collected data (Knoppers and Beauvais, 2021). From the perspective of donors, ensuring data safety is of particular importance. According to the study by Makhlouf et al. (2019), as many as 97% of all respondents admit that the incentive factor for donating biological material to a biobank was the awareness that measures to ensure privacy and confidentiality will be applied. In other studies, respondents were more willing to donate a sample if they had knowledge about who would have access to the results and where and how the samples would be stored (Goodson and Vernon, 2004). An important factor in building trust is the anonymization of samples, as well as the ability to withdraw from participation in the study. An important ethical issue concerns incidental and secondary findings from genomic sequencing. These are divided into the following three main groups: (a) clinically actionable results, (b) results of clinical significance for disease but usually not expected to be clinically actionable, and (c) results of little or no clinical significance for disease and harm (Berg, Khoury, Evans, 2011). The majority of studies conducted in high-income countries (HICs) have shown that participants generally express a preference to receive feedback on their individual results; however, which genetic results participants would like to receive and why is not clear, and researchers should take into account the participants’ context, relationships, and empowerment in interpreting their preferences regarding feedback on results (Ralefala et al., 2021). Few guidelines have been developed outlining procedures for providing feedback on genomic research results in an ethical and legally compliant manner (Aizawa et al., 2020; West et al., 2020; Lewis, Knoppers, Green, 2021). Researchers should consider plans for providing participants with feedback on actionable genomic research results at the project proposal stage, obtain appropriate informed consent, ensure that resources have been allocated, and provide sufficient capacity and expertise to effectively support the feedback process (Matimba et al., 2022). Among the factors motivating to becoming an HBM donor, it is worth noting the possibility of learning the results of scientific research in which the sample will be used. In this way, participants would have a tangible opportunity to see how their samples contributed to the advances in medicine. Similar results were obtained in the work of Heredia et al. (2017) where respondents attached great importance to the knowledge about the way their samples were used. In the work of Merdad et al. (2017) 44% of the respondents believed that the donation of biological material would contribute to the progress of medical research and bring benefits to the society. Therefore, a transparent policy of information management, on the projects carried out and the results of research in which the resources of biobanks are used, constitutes an important motivating factor. It is worth adding that undoubtedly the information about the development of a specific diagnostic or therapeutic method is of greater interest than solely the information about the scope and type of conducted research. It is known that potential research participants are interested in increased control over how their tissue is used and desire methods for ongoing involvement (Lensink et al., 2022). Therefore, a new form of informed consent in the context of biobanking is a highly debated ethical and social issue. To maintain continuous engagement with research participants, new models of consent are proposed, such as blanket consent (pertaining to the process wherein individuals provide their samples without any restrictions), broad consent (pertaining to the process wherein individuals provide their samples for a wide range of unspecified future research with certain limitations), dynamic consent (digital decision support, wherein modern IT communication strategies are utilized to continuously inform and offer donors choices to determine the types of research their samples can be used for), or tiered consent (research can be categorized into levels or tiers, and participants can specify the types of research their samples will be used for) (Budin-Ljøsne et al., 2017; Domaradzki and Pawlikowski, 2019; De Sutter et al., 2022; Wiertz and Boldt, 2022).

The type of a biobank is an additional factor encouraging the donation of biological material. The majority of respondents expressed greater confidence in public institutions, including those particularly located within the organizational structure of a university or hospital, while maintaining a distance (sometimes differentiated by gender) from private institutions. The distance and even negative assessment towards the commercialization of human biological material and cooperation with private entities is also revealed in other studies. Many respondents point to concerns about the commercial use of samples, value public biobanks more than private ones and are reluctant to donate their material to commercial entities (Critchley et al., 2015; Farrugia et al., 2015; Dive et al., 2020).

Differences in the functioning of private and public biobanks extend beyond merely the commercial nature of their activities. Private biobanks exhibit more dynamic growth, greater involvement, and interest in effective communication with potential participants. Additionally, genetic research findings from private biobanks are typically available more expeditiously than from public ones, where timing is determined by the execution of specific research projects. Simultaneously, private biobanks do not receive public funding, have significantly less access to additional data and research infrastructure, prioritize ethical, social, and legal issues [ELSI] related to biobanking to a lesser extent, and ultimately encounter significantly less public trust (Tozzo and Caenazzo, 2020), as confirmed by the results of our study.

The issue of the material benefits of donation, including the reimbursement of costs related to participation in the research and the justification for remuneration for donors of biological material, is also topical for the discussion of biological material donation. On the one hand, it is ethically desirable to assume an altruistic attitude of donors (and such an attitude is often declared by donors). On the other hand, the commercialisation of human biological material and derived products may raise concerns from the perspective of the principles of social justice and donor protection (Knoppers et al., 1999; Check, 2006; Lenk and Beier, 2012). Furthermore, as noted by Pawlikowska et al. (2023), many links in the chain of products derived from Human Biological Materials (HBM) are not subject to principles of altruism or non-profit. Various entities, apart from the donor, may obtain material benefits. Therefore, it seems reasonable to consider permitting compensation for the donation of material, particularly for research and production purposes in the pharmaceutical and biotechnological industry (Pawlikowska et al., 2023). The matter of remuneration for the donation of biological material for research purposes is not unequivocally clear and raises lively discussion both in theoretical field and in biobanking practice. In reality, remuneration may entail risks of abuse or lead to decisions that would not have been made without financial compensation. However, the question arises as to whether even nominal remuneration to the donor is prohibited. In practice, various forms of remuneration for the donation of biological material, such as hair or blood, exist. Financial remuneration for costs, risks, or losses associated with participation in a biobank is also permissible (Pawlikowska et al., 2023).

In our study, as many as 70% of respondents were of the opinion that remuneration should be provided for the donation of samples to a biobank, and only less than every 10th Pole had the opposite opinion. Furthermore, among the factors motivating the donation of biological material, a small financial remuneration was indicated by more than 64% of the respondents. Such answers therefore indicate that this is an ambiguous issue that may raise many controversies, but at the same time it requires the attention and interest of biobanks and their managers.

In the research by Heredia et al. (2017) respondents openly admitted that monetary benefits would contribute to an increase in the willingness to donate biological material, which, to some extent, finds its confirmation in the results of our own research. In turn, in the work of Igbe and Adebamowo (2012) carried out on the Nigerian population, material benefits were rarely mentioned as a significant incentive to donate biological material, however, the possibility of obtaining healthcare in the event of detecting a disease during the research was indicated as an important factor motivating donation.

In the light of our research, it seems that material benefits may be an important factor shaping the attitudes of donors, but they are not as important as intangible benefits in the form of access to knowledge about one’s health, genealogical relationships, or the results of research based on the donated sample. Undoubtedly, the issue of rewarding research participants, including donors of biological material, requires in-depth ethical, legal and social analyses in the future, especially in the context of the commercialisation of research on human biological material.

The limitations of our research result from the impossibility to directly extrapolate the obtained research results to other populations due to potential cultural differences. However, consistency with other research results and the study involving a large and representative group allow treating the obtained results as reliable. The application value of the obtained research results is not universal, due to the diverse organizational and financial capabilities of biobanks, but it may provide some indication for the formulation of a catalogue of incentives for potential donors of biological material.

There are numerous issues that current and future biobanks must address. Several examples among many others analyzed in the latest literature on biobanking include material transfer agreements, access to samples and data, ownership and custodianship of data and samples, feedback regarding results and incidental findings (Caenazzo, Tozzo, 2020), big data, and artificial intelligence in biobanking (Kinkorová and Topolčan, 2020; Tozzo et al., 2023). All these issues need to be taken into consideration when planning future research.

## 8 Conclusion

One of the strongest factors that encourage cooperation with a biobank is access to genetic tests - including predisposition to diseases and genetic ancestry tests, as well as additional laboratory analyses.

A slightly smaller but also positively important role is played by the location of the biobank being within the organisational structure of a hospital or university, as well as the existence of a transparent information policy concerning the use of samples and the results of scientific research carried out on samples.

Material incentives in the form of compensation or reimbursement of costs play a less significant role.

A factor reducing the willingness to cooperate is the private status of the biobank and its commercial activity.

The organisation and management of biobanks and other entities conducting research on human biological material should take into account factors that may have a significant impact on the effectiveness of cooperation with research participants.

## Data Availability

The original contributions presented in the study are included in the article/Supplementary Material, further inquiries can be directed to the corresponding author.
